# Multiscale Freestanding Vertically Aligned CNT Heat Sink Enabling Multimodal Heat Dissipation by Conduction, Convection, and Radiation

**DOI:** 10.1002/advs.78540

**Published:** 2026-11-03

**Authors:** Jongwon Park, Kwangjun Kim, Jimin Lee, Jong G. Ok

**Affiliations:** ^1^ Department of Automotive Engineering Seoul National University of Science and Technology Seoul Republic of Korea; ^2^ School of Mechanical Systems Engineering Seoul National University of Science and Technology Seoul Republic of Korea

**Keywords:** heat sink, multimodal heat dissipation, multiscale structure, thermal management, vertically aligned carbon nanotube

## Abstract

A vertically aligned carbon nanotube (VACNT)‐based heat sink is demonstrated for passive multimodal thermal management applicable for electronic and semiconductor devices. The VACNT enables multimodal heat dissipation through axial conduction, convection facilitated by its nanoporous morphology, and radiation enabled by its superior emissivity. While conventional metallic heat sinks suffer from low surface emissivity and require complex post‐processing to enhance radiative cooling, the proposed VACNT overcomes these limitations by providing an inherent multiscale structural hierarchy without secondary surface treatments. The thermal conductivity of the freestanding VACNT (500 to 1500 µm heights) is characterized using laser flash analysis and differential scanning calorimetry as 32 to 40 W/mK. Evaluated on a Raspberry Pi Zero 2 W platform, the 1500 µm‐high VACNT heat sink decreases the peak CPU temperature by 10.7°C compared with a bare CPU. The VACNT can be patterned into the topographic architecture to further improve heat dissipation by increasing the surface area, achieving a 6.7°C lower peak CPU temperature than a conventional aluminum heat sink. Cyclic stability test confirms consistent thermal performance over repeated use. The VACNT practically allows various configurational engineering by molding, rolling, scissoring, and tiling, showcasing its versatility for diverse thermal management applications.

## Graphical abstract

This study demonstrates a vertically aligned CNT (VACNT)‐based heat sink. The nanoporous structure of the VACNT provides a large surface area for convective and radiative exchange, alongside axial conduction, enabling multimodal heat dissipation. Mounted on a CPU, the VACNT with a fin array pattern achieves a steady‐state temperature 6.7°C lower than a conventional aluminum heat sink.

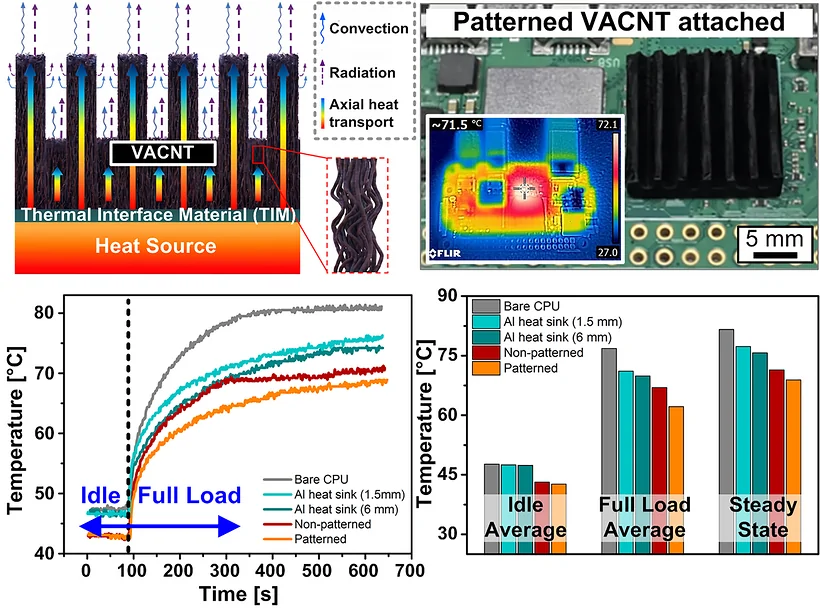

## 1 Introduction

Recently, rapid advancement of high‐performance computing and artificial intelligence (AI) technologies has significantly intensified heat generation in diverse electronic and semiconductor devices [1, 2, 3, 4, 5, 6], which makes thermal management one of the most critical bottlenecks limiting device performance, reliability, and operational lifespan [7, 8, 9, 10]. Conventional heat sink materials with high thermal conductivity, such as copper (∼400 W/mK) and aluminum (∼200 W/mK), have been widely adopted due to their mature fabrication processes. However, their inherently low surface emissivity (ε ≈ 0.03 for polished copper and ε ≈ 0.05 for polished aluminum) limits radiative heat dissipation to the surroundings, which is a dominant mechanism for effective heat dissipation [11, 12, 13]. Such systems must efficiently conduct heat away from the source while simultaneously dissipating it through convection and radiation to maximize overall thermal performance [8, 14, 15]. Specifically, next‐generation electronic systems strongly require multimodal heat dissipation‐enabled materials and structures [16].

Vertically aligned carbon nanotube (VACNT) has attracted considerable attention as a superb thermal management material [17, 18, 19, 20, 21], owing to the inherent extraordinary axial thermal conductivity [22, 23, 24, 25]. Furthermore, the vertically aligned structure enables efficient heat conduction along the axial direction of the CNTs, facilitating rapid heat transfer from a heat source [26, 27]. In addition, the VACNT exhibits high emissivity (ε ≈ 0.98) arising from its nanoscale porous structure [28, 29]. This nanoscale porosity and the resulting superior emissivity are intrinsic to the VACNT structure, requiring no additional surface treatment or functionalization. The nanoscale porosity between individual CNTs further provides a large effective surface area for convective and radiative heat exchange [30, 31]. While conventional metallic heat sinks offer limited emissivity and lack this multiscale structural hierarchy [32, 33], the VACNT, configurable into a variety of multiscale forms (e.g., millimeter‐high freestanding pad), can be utilized as a multimodal heat sink.

Despite these promising advantages, practical utilizations of VACNT as a heat sink are still challenging. The inherently weak mechanical integrity of VACNT makes controlled delamination from the growth substrate difficult without structural damage. Moreover, transferring the delaminated VACNT onto a target surface while maintaining its vertical alignment and nanoporous structure, also remains a vivid challenge [34, 35, 36]. However, once these issues are properly addressed, the direct application of the freestanding VACNT as a high‐performance heat sink component can come true.

In this study, we investigate the multimodal heat dissipation characteristics of the freestanding VACNT obtainable from the controlled catalyst oxidation during the CVD growth process [37]. This freestanding VACNT exhibits an inherent multiscale structure. Its millimeter‐scale height provides an efficient axial conduction pathway, enabling its direct use as a heat sink. Its nanometer‐scale porosity simultaneously offers a large effective surface area and a superior emissivity, enabling multimodal heat dissipation through conduction, convection, and radiation within a single material. The thermal properties of the freestanding VACNTs of various growth heights of 500 to 1500 µm are systematically characterized as a function of growth height using laser flash analysis (LFA) and differential scanning calorimetry (DSC). Upon evaluated as a heat sink on a Raspberry Pi Zero 2 W platform, the VACNT heat sink achieves effective heat dissipation with a ∼10°C lower peak steady‐state CPU temperature compared to bare CPU operation. To further enhance the thermal performance by increasing the surface area, the VACNT is patterned via mold pressing method into a fin‐array geometry, achieving a 6.7°C lower peak CPU temperature than a commercial aluminum heat sink. This patterned VACNT heat sink also exhibits stable thermal performance over repeated thermal cycles, confirming its reliability for practical operation. It is also demonstrated that VACNT can be patterned, rolled, scissor‐cut, and tiled into freeform scalable architectures, further highlighting its broad practical applicability to diverse thermal management environments.

## 2 Results and Discussion

### 2.1 Fabrication Process and Structural Properties of the VACNT

The overall fabrication process of the freestanding VACNT is illustrated in Figure 1. First, the VACNT was grown by using chemical vapor deposition (CVD) on a SiO_2_ substrate pre‐deposited with a 2 nm‐thick Fe layer on a 10 nm‐thick Al_2_O_3_ (Figure 1a). Scanning electron microscopy (SEM) images of the annealed catalyst layer before CNT growth shows Fe nanoparticles with an average diameter of 17.3 nm and an areal density of 7.44 × 10^10^ /cm^2^ (Figure S1a,b). Each nanoparticle serves as an individual nucleation site for a single CNT, and the areal density of the catalyst layer thus directly determines the packing density of the resulting VACNT. Notably, both the packing density and growth height of the VACNT are expected to significantly influence their thermal properties. Therefore, all CVD parameters, including the temperature and duration of annealing and growth step, were kept as fixed conditions throughout the experiments, while only the CNT growth time was varied to mainly control the growth height. We have controlled the growth height of the VACNT by adjusting the duration of C_2_H_4_ supply (carbon‐source gas supplying time) during the CVD process to evaluate the thermal performance based solely on the growth height. The resulting growth heights of 109.41±3.71, 503.24±7.35, 1006.37±5.17, and 1586.79±8.67 µm are achieved with the growth times of 2, 8, 15, and 60 min, respectively, on the Fe/ Al_2_O_3_‐coated SiO_2_ substrate of the area of 1.5 × 1.5 cm (Figure 1g). The detailed setup and conditions for the CVD process for CNT growth can be found in the literature [38, 39, 40, 41, 42]. Interestingly, the relationship between growth time and growth height is not strictly linear, as the growth rate gradually slows with increasing growth time. This is attributed to the progressive deactivation of catalyst nanoparticles during prolonged growth, which reduces the number of active nucleation sites and leads to a gradual increase in structural defects within the CNT walls [43, 44]. Its implications on thermal properties are discussed in Section 2.2.

**FIGURE 1:**
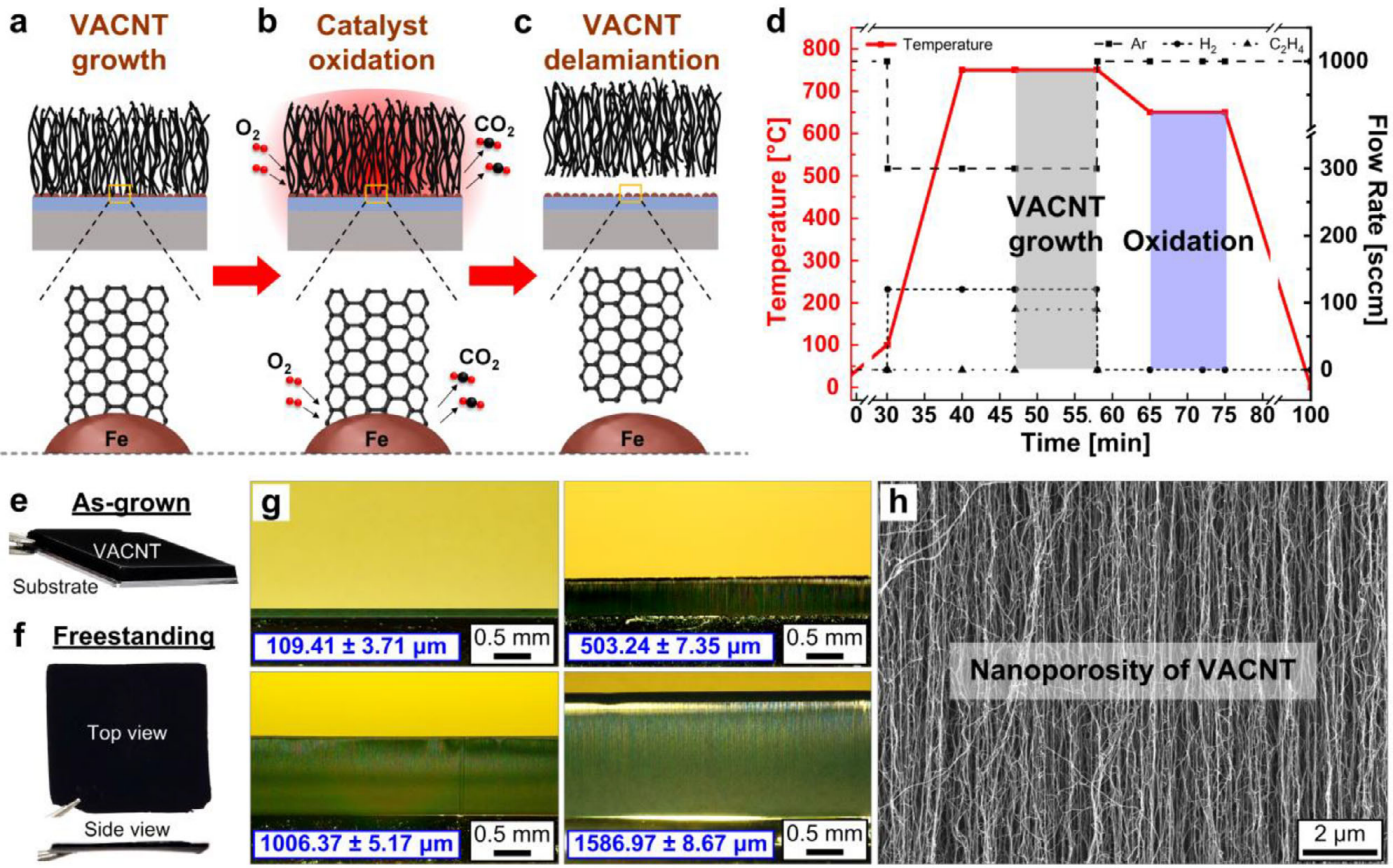
(a–c) Schematics for the fabrication procedure of the freestanding VACNT. (d) CVD recipe for the VACNT growth and catalyst layer oxidation. Optical images of (e) the as‐grown VACNT on the SiO_2_ substrate and (f) the freestanding VACNT (top and side views). (g) Optical images (side views) of the as‐grown VACNT samples with various growth heights. (h) SEM image (cross‐sectional view) showing the nanoporosity of the freestanding VACNT.

Following VACNT growth, oxidation of the catalyst layer was performed to delaminate the VACNT from the SiO_2_ substrate (Figure 1b,c) [37]. The VACNT is grown via a bottom‐up mechanism in which carbon atoms are adsorbed onto the catalyst layer, dissolve into catalyst nanoparticles, and subsequently precipitate to form graphitic hexagonal rings that extend upward [45, 46]. However, the carbon atoms at the VACNT‐catalyst interface form structurally incomplete hexagonal rings due to their bonding constraints with the underlying catalyst layer, rendering them chemically less stable than those in the bulk CNT walls. Upon exposure to an Ar/air atmosphere at 600°C, oxygen preferentially oxidizes these interfacial carbon atoms, releasing them as CO_2_. As a result, the adhesion between the VACNT and the substrate is progressively weakened, enabling delamination. Meanwhile, as the growth height increases, oxygen penetration to the catalyst layer becomes increasingly hindered, thereby requiring a longer oxidation time to achieve complete delamination. Accordingly, the oxidation duration was adjusted from 15 to 30 min depending on the growth height.

As shown in Figure 1f, the delaminated VACNT maintains its freestanding form without the substrate's support. This makes it readily handleable and directly transferable onto target devices for thermal management. Figure 1h shows a cross‐sectional SEM image of the freestanding VACNT, which confirms that the nanoporous structure intrinsic to the VACNT is well preserved after delamination. As will be discussed in detail in the following sections, such a vertically aligned nanoporous structure is greatly favorable for multimodal heat dissipation. Axial conduction along the vertically aligned CNT architecture [20, 22, 23, 24], convection facilitated by the large effective surface area of the nanoporous morphology, and radiation enabled by the widely reported high emissivity (ε ≈ 0.98) of the nanoporous surface [28, 29], collectively make the freestanding VACNT highly efficient for passive thermal management. Despite this potential, prior studies on VACNT‐based thermal management have mainly explored two limited configurations. In one approach, the VACNT has been employed as a thin thermal interface material (TIM) inserted between a heat source and a conventional heat sink, where the CNTs are grown to a short height and serve primarily to reduce the interfacial contact resistance rather than to function as a heat‐dissipating structure [47, 48, 49]. In the other approach, the as‐grown VACNT has been infiltrated with a polymer matrix to form composite structures on the substrate to facilitate handling and delamination [25, 50, 51]. This infiltration, however, inevitably fills the nanoporous network between individual CNTs, thereby compromising both the nanoporosity and resultant emissivity, which consequently limits the achievable radiative and convective heat dissipation performance. As a result, direct utilization of the VACNT retaining their freestanding, nanoporous architecture as a standalone heat sink, aiming for combined conductive, convective, and radiative heat dissipation, remain largely unexplored.

### 2.2 Thermal Properties of the Freestanding VACNT

Notably, due to the highly directional heat conduction along the *sp* ^2^ carbon bonds of individual CNTs and their macroscopic vertical alignment, the VACNT exhibits pronounced thermal anisotropy. As the thermal properties are dominant along the CNT axis, characterizing the thermal conductivity (κ) in the longitudinal direction is critical for evaluating the overall thermal performance. To this end, laser flash analysis (LFA) and differential scanning calorimetry (DSC) were performed in the longitudinal direction on the VACNT samples with the growth heights of 500, 1000, and 1500 µm (simply referred to as the 500 µm VACNT, 1000 µm VACNT, and 1500 µm VACNT, respectively, hereafter). The 100 µm VACNT was excluded from this characterization, as its thickness was below the practical measurement limit of the LFA instrument used in this study. Due to the difficulty in direct measurement of thermal conductivity, it is calculated using the following equation:

(1) $$\kappa =\alpha \cdot \rho_{\mathrm{bulk}}\cdot C_{p}$$

where α is the thermal diffusivity measured by LFA, ρ_bulk_ is the bulk density of the VACNT, and C_p_ is the specific heat capacity. ρ_bulk_ of the VACNT was directly determined from the measured mass and volume of each sample; here to ensure the measurement trustworthiness, up to six VACNT samples grown to a height of 1 mm on the 1.5 × 1.5 cm substrates were prepared and their averaged mass (∼87.0 mg) and volume (calculated by its dimensions) were used (Figure S1c–e); ρ_bulk_ yields a value of 386.5 mg/cm^3^. C_p_ is obtained by DSC using sapphire as a reference standard (Figure S2), giving a value of 1.254 J/gK at 25°C. Figure 2a,b presents the measured thermal diffusivity and the resulting thermal conductivity, respectively, for the VACNT samples with various heights. Interestingly, the thermal diffusivity decreases from 83 mm^2^/s (500 µm VACNT) through 77 mm^2^/s (1000 µm VACNT) to 66 mm^2^/s (1500 µm VACNT). As thermal conductivity is directly proportional to thermal diffusivity (Equation (1)), with ρ_bulk_ and C_p_ remaining constant across samples, thermal conductivity follows the same trend, decreasing from 40 W/mK (500 µm VACNT) through 37 W/mK (1000 µm VACNT) to 32 W/mK (1500 µm VACNT). To elucidate this relation between the structural and materials characteristics, Raman spectroscopy was performed for the 500, 1000, and 1500 µm VACNT samples (Figure 2c). Two characteristic Raman peaks were observed: D peak at 1350 cm^−1^ and G peak at 1580 cm^−1^. The G peak is associated with the in‐plane stretching vibration of sp^2^‐bonded carbon atoms in a well‐ordered hexagonal lattice, and therefore reflects the degree of graphitic ordering within the CNT walls. In contrast, the D peak originates from structural disorder and defects, such as vacancies, distorted hexagonal rings, and amorphous carbon. Consequently, the G/D intensity ratio is widely used as an indicator of CNT crystallinity, where a higher G/D ratio corresponds to lower defect density and improved structural quality [52, 53, 54, 55]. The measured G/D ratios for the 500, 1000, and 1500 µm VACNTs are 1.75, 1.67, and 1.53, respectively, exhibiting a gradual decrease with increasing growth height.

**FIGURE 2:**
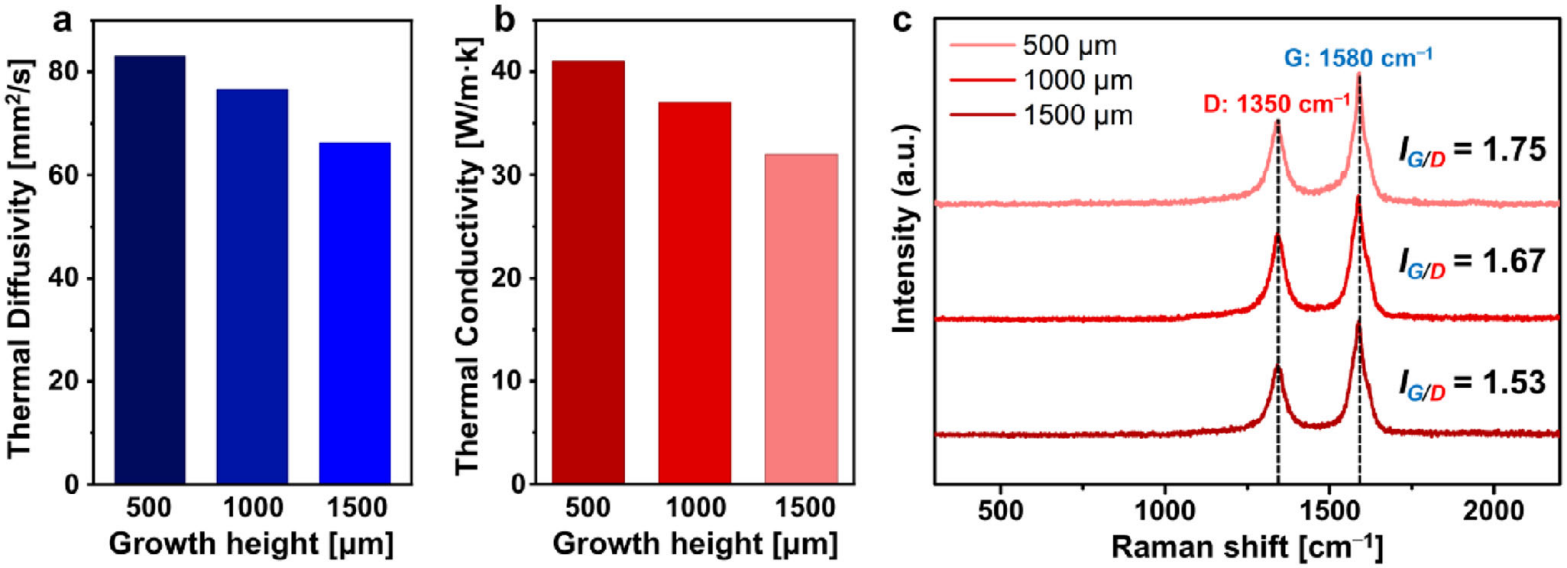
(a) Thermal diffusivity measured by laser flash analysis (LFA) and (b) thermal conductivity of the VACNTs with the growth heights of 500, 1000, and 1500 µm, calculated from Equation (1). (c) Raman spectra of the VACNTs with the growth heights of 500, 1000, and 1500 µm, with the corresponding D peaks (1350 cm^−1^) and G peaks (1580 cm^−1^) marked.

This gradual drop in G/D ratio with increasing growth height provides insight into why and how the structural change affects α and κ, particularly in correlation with structural defects. Specifically, structural defects are prone to accumulate within the CNT walls as growth time extends, presumably driven by two concurrent mechanisms. One contributing factor is the progressive deactivation of catalyst nanoparticles caused by amorphous carbon encapsulation and thermally induced sintering, which leads to non‐uniform carbon incorporation and incomplete graphitic structures [43, 56]. As another aspect, as the VACNT height increases for a longer growth time, the diffusion of the carbon source gas toward the catalyst layer can be obstructed due to a longer path [57, 58]. Defect formation thus becomes more pronounced, particularly in the upper regions of the VACNT. This increased defect density intensifies phonon scattering along the CNT axis. Because heat conduction in CNTs is primarily governed by phonon transport [22, 23], enhanced phonon scattering reduces the mean free path of phonons, thereby lowering α and leading to an overall decrease in κ with increasing growth height [21, 59].

Alongside this crystallinity‐thermal property relationship, the measured thermal conductivity values of 32–40 W/mK remain viable for heat sink applications when combined with the multimodal heat dissipation capability arising from the nanoporous structure and superior emissivity of the VACNT. These thermal properties, taken together, motivate its tangible application as a heat sink component, as explored in the following section.

### 2.3 Thermal Performance of the VACNT Heat Sink

To evaluate the thermal performance of the freestanding VACNT as a heat sink, it was assembled onto a Raspberry Pi Zero 2 W following the fabrication process illustrated in Figure S3. A MT224 (MAXTEK, Korea) thermal pad, used as a TIM, was first attached to the CPU of a Raspberry Pi Zero 2 W (Figure S3a). Then the freestanding VACNT is transferred onto the TIM to serve as a heat sink (Figure S3b). A compressive force of 5 N was applied during the transfer process to improve contact between the VACNT and the TIM layer without collapsing the nanoporous CNT structure. To further enhance the thermal performance by increasing the surface area, the VACNT could be additionally patterned by mold pressing using a 3D‐printed mold to form a fin array geometry (Figure S3c). The effect of this patterning on the thermal performance of the VACNT heat sink is discussed later in Section 2.4.

Now, we first investigate the thermal performance of the non‐patterned VACNT heat sink as a function of growth height by monitoring the CPU temperature under full‐load operation (all CPU cores fully utilized via stress test) for the 100, 500, 1000, and 1500 µm VACNT application cases, in comparison with the bare CPU as a control. Basically, heat generated from the CPU is first conducted axially through the VACNT, which serves as the primary conductive pathway due to its vertically aligned structure. Then, the transported heat is dissipated to the surrounding environment through two pathways: convection and radiation from the whole VACNT structure (Figure 3a); the millimeter‐scale VACNT enables facile axial conduction and provides sufficient surface for convective heat exchange, while its superior emissivity (ε ≈ 0.98) boosts efficient radiative dissipation.

**FIGURE 3:**
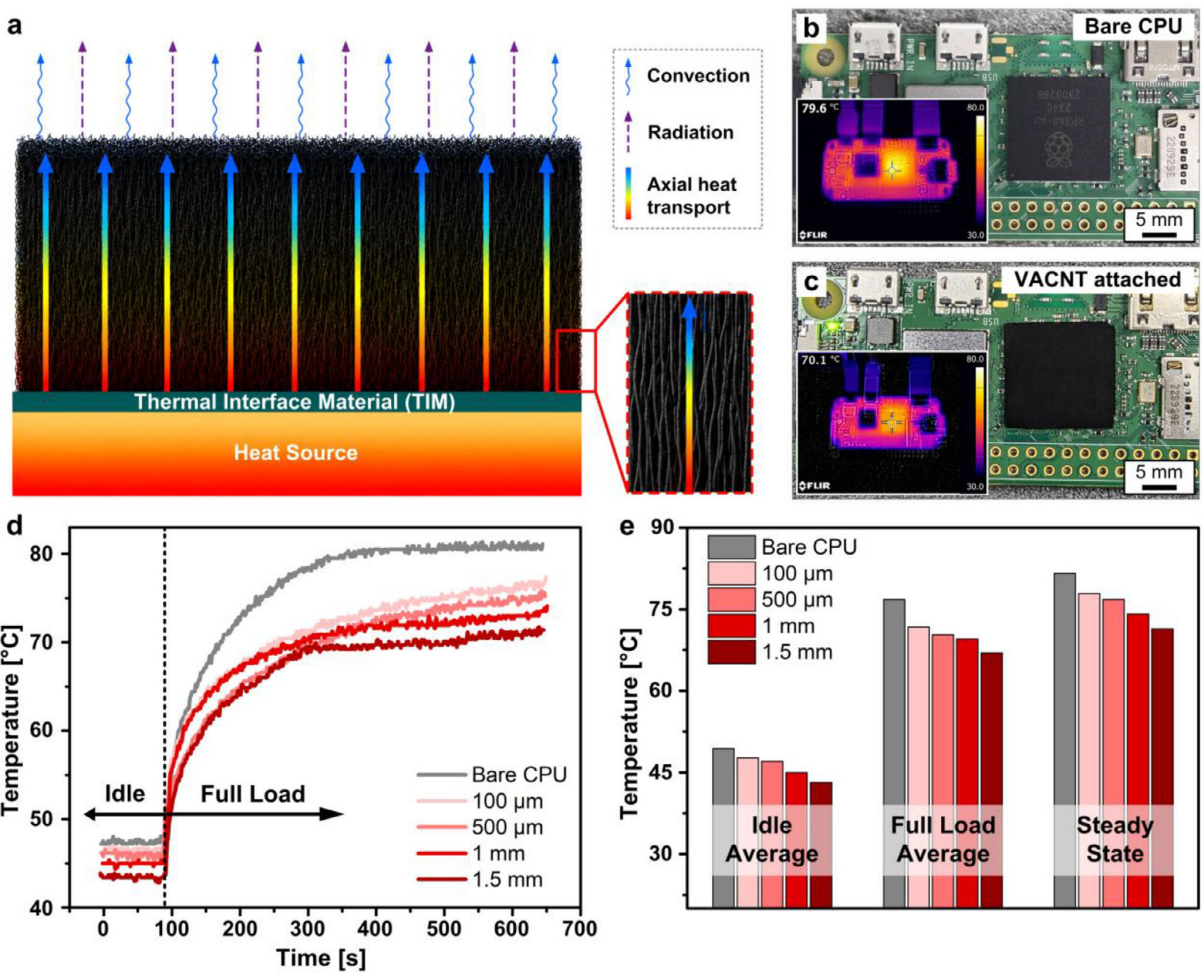
(a) Illustration of the heat dissipation pathways in the VACNT heat sink. Optical and infrared (inset) images of the Raspberry Pi with (b) a bare CPU and (c) a 1500 µm VACNT heat sink under full load conditions. (d) CPU temperature profiles and (e) summary of CPU temperatures under idle and full load conditions for the bare, 100, 500, 1000, and 1500 µm VACNT‐applied cases.

Based on these multimodal heat dissipation mechanisms, we have investigated the CPU temperature profiles from idle to full load and steady‐state conditions for the bare and 100, 500, 1000, and 1500 µm VACNT heat sinks‐applied cases (Figure 3d,e). Upon the application of full load, the CPU temperature rises and eventually converges to a steady‐state temperature for each condition. This steady‐state temperature serves as a direct indicator of the heat sink's thermal performance, as a lower steady‐state temperature reflects more effective heat dissipation. Specifically, the steady‐state CPU temperature decreases with increasing growth height: 77.4°C, 76.3°C, 73.6°C, and 70.9°C for 100, 500, 1000, and 1500 µm VACNTs, respectively, compared to 81.6°C for the bare CPU. Consistent with the CPU temperature measurements, the infrared thermal images in Figure 3b,c shows a lower peak surface temperature for the 1500 µm VACNT heat sink (70.1°C) than for the bare CPU (79.6°C) under identical full‐load conditions.

To quantify the thermal performance, we decompose the total thermal resistance from the CPU to ambient into two physically distinct components: the thermal resistance from the CPU to the TIM (R_CPU‐TIM_) and the thermal resistance from the TIM to the ambient air through the VACNT heat sink (R_TIM‐air_). At each surface, the heat flux dissipated to the ambient by combined natural convection and radiation is given by:

(2) $$Q_{conv}=A\cdot h_{conv}\cdot \left(T_{s}-T_{amb}\right)$$

(3) $$Q_{rad}=A\cdot \varepsilon \cdot \sigma \cdot \left(T_{s}^{4}-T_{amb}^{4}\right)$$

where A is the surface area, T_s_ is the surface temperature, T_amb_ is the ambient temperature (25°C), ε is the surface emissivity, σ is the Stefan‐Boltzmann constant, and h_conv_ is the natural convection heat transfer coefficient. Because the measurements were performed indoors under continuous air circulation from the air‐conditioning system rather than under stagnant‐air conditions, h_conv_ = 20 W/m^2^K was assumed as a representative convective heat‐transfer coefficient for the experimental environment. The total heat dissipated from the surface is the sum of the two contributions:

(4) $$Q_{total}=Q_{conv}+Q_{rad}$$

First, to determine R_CPU‐TIM_, the TIM was attached to the CPU, leaving the TIM surface directly exposed to ambient air. The CPU temperature (T_cpu_) and the TIM surface temperature were measured under the full‐load steady state. The heat dissipated from the exposed TIM surface (Q_TIM_) was calculated using Equations ((2), (3), (4)) with the TIM surface area (A_TIM_ = 1.5 × 1.5 cm^2^) and emissivity (ε_TIM_ = 0.90). R_CPU‐TIM_ is calculated as:

(5) $$R_{CPU-TIM}=\frac{\left(T_{CPU}-T_{TIM,surface}\right)}{Q_{TIM,total}}$$

The obtained values are summarized in Table 1. Infrared thermal images corresponding to this measurement are provided in Figure S4.

**TABLE 1:** Thermal resistance of the CPU‐TIM interface (T_ambient_ = 25°C, h_conv_ = 20 W/m^2^K, ε_TIM_ = 0.90).

Condition	T_cpu_ [°C]	T_TIM, surface_ [°C]	Q_conv_ [W]	Q_rad_ [W]	Q_TIM, total_ [W]	R_CPU‐TIM_ [°C/W]
TIM only	77.3	75.3	0.226	0.078	0.305	6.56

The small value of R_CPU‐TIM_ is consistent with the intended role of the TIM; rather than serving as a heat‐dissipating element, the TIM functions solely as an interface layer that minimizes contact resistance between the CPU and the heat sink. Overall, the low R_CPU‐TIM_ confirms that the TIM plays this role efficiently and does not constitute a bottleneck in the overall heat‐transfer pathway.

Now we quantitatively examine how the increase in VACNT sidewall area with growth height contributes to the enhanced thermal performance. Q_conv_ and Q_rad_ were calculated for each growth height using the top and sidewall surface temperatures, from which R_TIM‐air_ was determined. For the VACNT heat sink, R_TIM‐air_ cannot be calculated directly because the TIM‐VACNT interface temperature (T_interface_) cannot be measured directly, as it is buried between the two solid layers. T_interface_ was therefore back‐calculated from the CPU temperature of the assembled system, using R_CPU‐TIM_ obtained above and the heat dissipated from the VACNT surface (Q_VACNT, total_) by:

(6) $$T_{interface}=T_{CPU}-R_{CPU-TIM}\cdot Q_{VACNT,total}$$

where Q_VACNT, total_ is the sum of the heat dissipated from the top surface (Q_top_) and the lateral sidewalls (Q_side_) of the VACNT, each calculated from Equations ((2), (3), (4)) using the respective infrared‐measured surface temperatures (T_top_ and T_side_), corresponding surface areas (A_top_ and A_side_), and the emissivity of the VACNT (ε_VACNT_ = 0.98). R_TIM‐air_ is calculated by:

(7) $$R_{TIM-air}=\frac{(T_{interface}-T_{amb)}}{Q_{VACNT,total}}$$

The resulting values for the 100, 500, 1000, and 1500 µm VACNT heat sinks are summarized in Table 2. For the 100 µm VACNT, the sidewall height was too small to resolve a reliable infrared signal; given that the sidewall area accounts for only ∼2.7% of the top‐surface area at this height, Q_side_ was neglected, and R_TIM‐air_ was calculated from Q_top_ alone. Infrared thermal images corresponding to this measurement are provided in Figure S4.

**TABLE 2:** Thermal resistance of the TIM‐to‐ambient surface for the VACNTs of various heights (T_ambient_ = 25°C, h_conv_ = 20 W/m^2^K, ε_VACNT_ = 0.98).

Condition	T_cpu_ [°C]	T_top_ [°C]	T_side_ [°C]	Q_conv, top_ [W]	Q_rad, top_ [W]	Q_conv, side_ [W]	Q_rad, side_ [W]	Q_VACNT, total_ [W]	R_TIM‐air_ [°C/W]
100 µm VACNT	77.4	75.5	—	0.2272	0.0859	—	—	0.3132	160.7
500 µm VACNT	76.3	74.4	75.1	0.2223	0.0836	0.0301	0.0113	0.3473	141.1
1000 µm VACNT	73.6	72.6	73.3	0.2142	0.0799	0.0580	0.0217	0.3737	123.5
1500 µm VACNT	70.9	70.1	70.8	0.2029	0.0748	0.0824	0.0305	0.3906	110.9

Consistent with measured temperatures, R_TIM‐air_ decreases monotonically from 160.7°C/W for the 100 µm VACNT to 110.9°C/W for the 1500 µm VACNT, indicating progressively improved heat dissipation at the VACNT surface with increasing growth height. R_TIM‐air_ is consistently one to two orders of magnitude larger than R_CPU‐TIM_, indicating that the overall system performance is governed almost by the heat‐exchange capability of the VACNT surface. Notably, the heat dissipation performance improves with increasing growth height, even though the thermal conductivity was previously shown to decrease over this range due to structural defects (40 W/mK for the 500 µm VACNT to 32 W/mK for the 1500 µm VACNT). This indicates that the reduction in thermal conductivity has only a limited influence on R_TIM‐air_. As the growth height increases, the lateral surface area of the VACNT sidewalls available for convective and radiative heat exchange grows proportionally; for a vertically aligned structure, this lateral area scales linearly with growth height, providing a larger interface for heat exchange with the surroundings. This trend is directly reflected in Q_side_, which increases from 0.041 W for the 500 µm VACNT to 0.080 W for the 1000 µm VACNT and further to 0.113 W for the 1500 µm VACNT, corresponding to an increase from approximately 12% to 21% and 29% of the total heat dissipated from the VACNT surface (Q_VACNT, total_), respectively. Consequently, the decrease in R_TIM‐air_ with increasing growth height reflects the fact that the VACNT‐height‐dependent reduction in thermal conductivity has only a limited impact on the overall resistance; instead, the proportional increase in the lateral sidewall surface area, and the resulting increase in its contribution to the total heat dissipation, efficiently enhances heat dissipation from the VACNT surface. Motivated by the strong influence of the surface area on thermal performance, we anticipate that further increasing the surface area, for instance by applying the patterning to the VACNT structure, would lead to even greater improvement in thermal performance.

### 2.4 Thermal Performance Enhancement via Patterning of the VACNT Heat Sink

Having evaluated the thermal performance of the VACNT heat sink with various growth heights, we study whether the increase in surface area could further improve the thermal performance. For this purpose, the 1500 µm VACNT, which has shown the highest thermal performance, was patterned by the mold pressing into a fin‐array geometry; hereafter, all patterned VACNT heat sinks refer to those fabricated from the 1500 µm VACNT. The geometry of the pressed area was designed to match the fin‐to‐base height ratio (2:1) of the commercial aluminum heat sink, enabling a closer comparison under the same topography. Specifically, the aluminum heat sink was evaluated both at its original height of 6 mm (4 mm fin, 2 mm base; hereafter Al heat sink (6 mm)) and after being machined down to match the height of the patterned VACNT (1.5 mm; 1 mm fin, 0.5 mm base; hereafter Al heat sink (1.5 mm)) in order to enable a comparison focused solely on the intrinsic material properties (Figure 4a–c). To determine the compressive force required to match the fin‐to‐base height ratio of the aluminum heat sink, the compressive behavior of the VACNT was evaluated using a universal testing machine. Based on the measured compression response (Figure S5), a force of 55 N was applied to achieve a fin‐to‐base ratio (2:1), resulting in a fin height of 1 mm and a base height of 0.5 mm. Notably, it is important to examine whether the intrinsic nanoporous structure was preserved after patterning, because the VACNT structure undergoes substantial compression during mold pressing. As shown in Figure 4e, localized buckling of individual CNTs was observed in the compressed regions, which is consistent with previously reported coordinated buckling behavior in compressed VACNT [60]. Nevertheless, the nanoporous structure intrinsic to the VACNT was largely preserved. This confirms that the compressed regions could continue to contribute to radiative and conductive heat dissipation.

**FIGURE 4:**
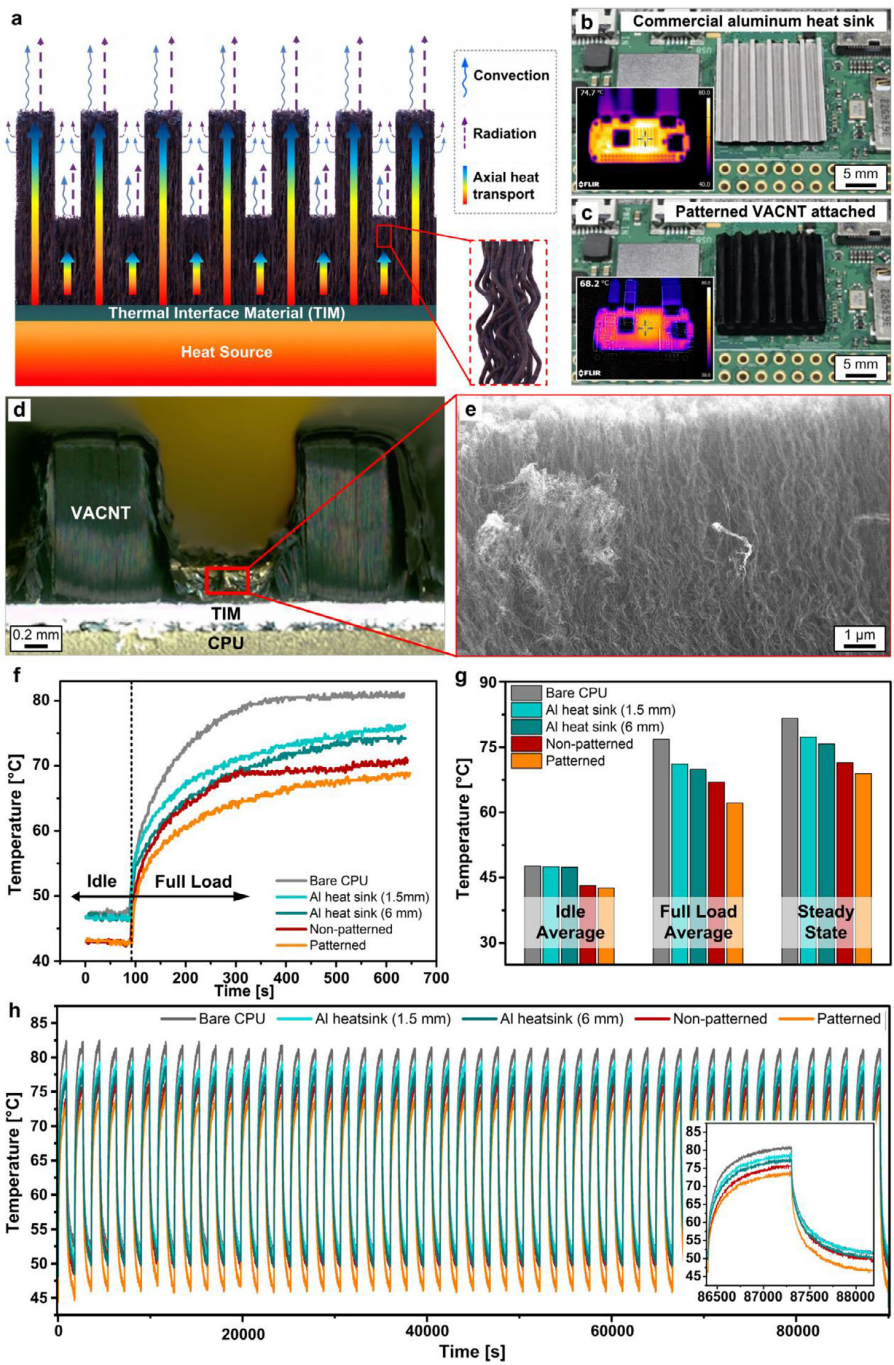
(a) Illustration of the heat dissipation pathways in the patterned VACNT heat sink. Optical and infrared (inset) images of the Raspberry Pi with (b) a commercial aluminum heat sink (6 mm height) and (c) a patterned VACNT heat sink under full load conditions. (d) Optical image (cross‐sectional side view) of the patterned VACNT. (e) SEM image (cross‐sectional view) of the mold‐pressed region of VACNT. (f) CPU temperature profiles and (g) summary of CPU temperatures under idle and full load conditions for the bare, Al heat sink‐applied (1.5 and 6 mm heights), and non‐patterned and patterned 1500 µm VACNT‐applied cases. (h) CPU temperature profiles of 50‐repeated thermal cycles for the corresponding cases.

Now we investigate the thermal performance of the patterned VACNT heat sink in direct comparison with a commercial aluminum heat sink as a control. To isolate the intrinsic material contribution from any geometric advantage, the patterned VACNT was first compared against the Al heat sink (1.5 mm), which shares an identical fin‐array geometry. As presented in Figure 4f,g, the patterned VACNT heat sink exhibits a steady‐state CPU temperature of 69.4°C, compared to 76.1°C for the Al heat sink (1.5 mm), under the same topography. This indicates that the temperature difference between the two configurations originates from their intrinsic material properties. The patterned VACNT heat sink further lowers the temperature by 1.5°C compared to the non‐patterned configuration, highlighting the benefit of the enhanced surface area. The patterned VACNT was also compared against the Al heat sink (6 mm), the original commercial heat sink with a larger surface area than either the patterned VACNT or the Al heat sink (1.5 mm). Under this comparison, the patterned VACNT heat sink still exhibited a lower steady‐state CPU temperature (69.4°C) than the Al heat sink (6 mm) (74.5°C).

Consistent with the CPU temperature measurements, the infrared thermal images in Figure 4b,c and Figure S6 shows a lower top‐surface temperature for the patterned VACNT heat sink (68.2°C) than for both the Al heat sink (1.5 mm) (74.7°C) and Al heat sink (6 mm) (73.1°C) under identical full‐load conditions. To quantitatively compare the relative contributions of convection and radiation to the overall heat dissipation, the top‐surface temperature of each configuration was used to calculate Q and R_TIM‐air_ using Equations ((2), (3), (4), (6), (7)), following the framework established in Section 2.3. Given the fin‐array geometry, incorporating the sidewall contribution into this comparison was limited by the difficulty of reliably measuring the temperature between closely spaced adjacent fins; the sidewall contribution was therefore excluded from the calculation, and the corresponding infrared images of the fin sidewalls are instead provided in Figure S6 for reference only. The resulting values are summarized in Table 3.

**TABLE 3:** Thermal resistances of the aluminum and patterned VACNT heat sinks (T_ambient_ = 25°C, h_conv_ = 20 W/m^2^K, A_top_ = 1.05×10^−4^ m^2^ for all configurations).

Condition	ε	T_cpu_ [°C]	T_top_ [°C]	Q_conv_ [W]	Q_rad_ [W]	Q_total_ [W]	R_TIM‐air_ [°C/W]
Al heat sink (1.5 mm)	0.05	76.1	74.7	0.1044	0.00201	0.1064	473.8
Al heat sink (6 mm)	0.05	74.5	73.1	0.1010	0.00193	0.1029	474.3
Patterned VACNT (1.5 mm)	0.98	69.4	68.2	0.0907	0.0331	0.1238	352.0

As shown in Table 3, R_TIM‐air_ of the patterned VACNT heat sink (352.0°C/W) is lower than that of both the Al heat sink (1.5 mm) (473.8°C/W) and Al heat sink (6 mm) (474.3°C/W), consistent with the CPU temperature comparison discussed above. This difference is primarily reflected in Q_rad_ which is over an order of magnitude larger for the patterned VACNT (0.0331 W) than for aluminum heat sinks (0.00201 and 0.00193 W for 1.5 and 6 mm ones, respectively), consistently attributed to the large emissivity difference between VACNT (ε ≈ 0.98) and aluminum (ε ≈ 0.05). In contrast, Q_conv_ is comparable across all three configurations, and even slightly lower for the patterned VACNT than for the aluminum heat sinks, reflecting its lower surface temperature. To examine whether the high emissivity of the VACNT alone accounts for this difference, the Al heat sink (6 mm) was additionally anodized to obtain a black‐anodized surface, which exhibits a high emissivity of approximately 0.90 [61, 62]. The black‐anodized Al heat sink (6 mm) exhibited a steady‐state CPU temperature of 72.1°C, which is 2.4°C lower than that of the normal Al heat sink (6 mm) (74.5°C), confirming the contribution of enhanced emissivity (Figure S7). However, this temperature still remained higher than those of the non‐patterned (70.9°C) and patterned VACNT heat sinks (69.4°C), indicating that the advantage of the VACNT cannot be attributed to its emissivity alone.

The superior thermal performance of the VACNT can therefore be explained by two factors: the radiative heat dissipation capability of the VACNT itself, and the additional effective surface area arising from its nanoporous structure. To first examine the radiative contribution in these two factors, an additional experiment was designed; Cu was conformally sputtered onto the 1500 µm VACNT surface for various deposition times of 5, 10, and 15 min (Figure S8). Because Cu possesses a much lower emissivity than VACNT (ε_Cu_ ≈ 0.03), progressive Cu deposition was expected to suppress radiative heat transfer by partially covering the nanoporous VACNT surface. Cross‐sectional SEM images confirm that the extent of Cu coverage increases markedly with deposition time; at 5 min (Figure S8c), the sputtered Cu forms only a thin conformal layer along individual CNT strands, leaving the nanoporous network largely open, whereas at 15 min (Figure S8d), the Cu deposition has become dense enough to almost completely fill the nanoporous structure. As shown in Figure S8e,f, the steady‐state CPU temperature increases progressively with increased Cu deposition time, reaching 75.1°C at 15 min deposition, which is substantially higher than that of the Cu‐undeposited 1500 µm VACNT (70.9°C). Notably, this degradation in thermal performance occurs although Cu deposition could improve, rather than worsen, the electrical contact characteristics of the VACNT. Sputtered metal coatings have been reported to bridge adjacent CNTs and partially fill inter‐tube gaps, which can lower the inter‐tube contact resistance [63, 64]. Despite this potentially favorable effect on contact resistance, the steady‐state CPU temperature increased progressively with Cu deposition time, indicating that the loss in radiative heat dissipation arising from the pronounced emissivity mismatch between Cu (ε ≈ 0.03) and VACNT (ε ≈ 0.98) and the progressive occlusion of the nanoporous surface appears to outweigh any contact‐resistance‐related benefit. These results provide further evidence that the radiative cooling capability of the VACNT is critically attributed to its nanoporous morphology and intrinsically superior emissivity.

The other contributing factor lies in the nanoporous structure of the VACNT itself. While the heat flux calculation discussed above captures the effect of surface emissivity, it does not account for the nanoscale interactions between the VACNT structure and the surrounding air. Physically, however, the opened nanoporous structure of the VACNT allows ambient air to penetrate into the inside of the structure and interact with the surfaces of individual CNTs, each of which contributes to heat dissipation. It significantly expands the effective heat exchange area far beyond what is captured in the calculated heat flux values, providing an additional pathway for enhanced thermal performance [17]. Furthermore, the anisotropic thermal conduction along the CNT axis facilitates efficient heat redistribution across the structure, potentially reducing local hot spots and lowering the overall thermal resistance [27, 65]. To verify the direct contribution of this nanoporous structure to the thermal performance, the VACNTs with distinctly different porosities were additionally fabricated by varying the catalyst annealing duration prior to growth. This approach yielded the VACNTs with low, normal (identical to the VACNT used throughout this work, Figure 1h), and high nanoporosities, respectively (Figure S9a–c), having the same growth height of 500 µm. The steady‐state CPU temperature decreased systematically with increasing nanoporosity, from 77.3°C for the low‐porosity VACNT to 74.5°C for the high‐porosity VACNT (Figure S9d,e). As all three samples share the same growth height and were evaluated under identical measurement conditions, this trend cannot be attributed to differences in geometric surface area or emissivity, and instead directly demonstrates that the extent of the nanoporous network available for air infiltration governs the additional heat‐dissipation pathway described above. Overall, these results suggest that the nanoporous VACNT structure provides additional heat‐dissipation pathways beyond those captured in the calculated heat flux values, underscoring the unique advantage of its multiscale architecture over conventional metallic heat sinks.

While the present study demonstrates the advantage of the VACNT heat sink over a conventional aluminum heat sink, we note that surface modification strategies could also be applied to enhance the emissivity and consequent radiative heat dissipation of a conventional aluminum heat sink while retaining its high thermal conductivity. Previous studies have explored such approaches, including CNT coating [66], surface etching [67], and anodic oxidation [68, 69] of aluminum heat sinks. These strategies, however, generally require additional post‐processing steps such as coating deposition, chemical etching, or electrochemical oxidation applied after the heat sink has already been fabricated. In contrast, the freestanding VACNT heat sink presented in this study is obtained simply by the CVD growth followed by transfer from the growth substrate, without requiring any additional surface treatment to achieve its high emissivity and nanoporous structure. We therefore do not claim that the freestanding VACNT architecture is the only means of achieving high radiative heat dissipation, but rather bring it out as a facile and practical route to combining high emissivity with a multiscale, patternable architecture.

Having established the superior thermal performance of the patterned VACNT heat sink under single‐cycle operation, its durability and reliability under repeated heating and cooling cycles were further evaluated to assess its practical viability for sustained operation. As shown in Figure 4h, all five configurations showed stable temperature profiles throughout all 50 cycles plotted herein. Furthermore, the patterned VACNT consistently maintains the lowest peak temperature among all configurations across every cycle, which verifies that the thermal performance of the patterned VACNT heat sink observed in the single‐cycle operation is sustainable under repeated thermal loading conditions. Such a durable and reliable performance highlights the potential of the VACNT heat sink for reliable operation in practical thermal management applications.

### 2.5 Practical Applicability of the VACNT Heat Sink in Various Forms

The mold‐pressing process demonstrated in the previous section is not limited to the fin geometry. By simply modifying the mold design, the VACNT can be patterned into a variety of architectures. Representative examples are shown in Figure 5a, where grid array and honeycomb‐patterned VACNTs were fabricated using the corresponding 3D‐printed molds. In both cases, the resulting patterns closely match the mold geometry, indicating that the process can be readily extended to other structural designs. Beyond 3D printing, the mold can also be fabricated by alternative methods such as CNC machining of metals or deep reactive ion etching (DRIE)‐involved MEMS processes, further expanding the range of achievable mold geometries and scales. Such a versatility in mold fabrication suggests that the fin geometry, scale, and pattern can be engineered to meet specific thermal management requirements, offering a high degree of design freedom in the development of the VACNT‐based heat sinks.

**FIGURE 5:**
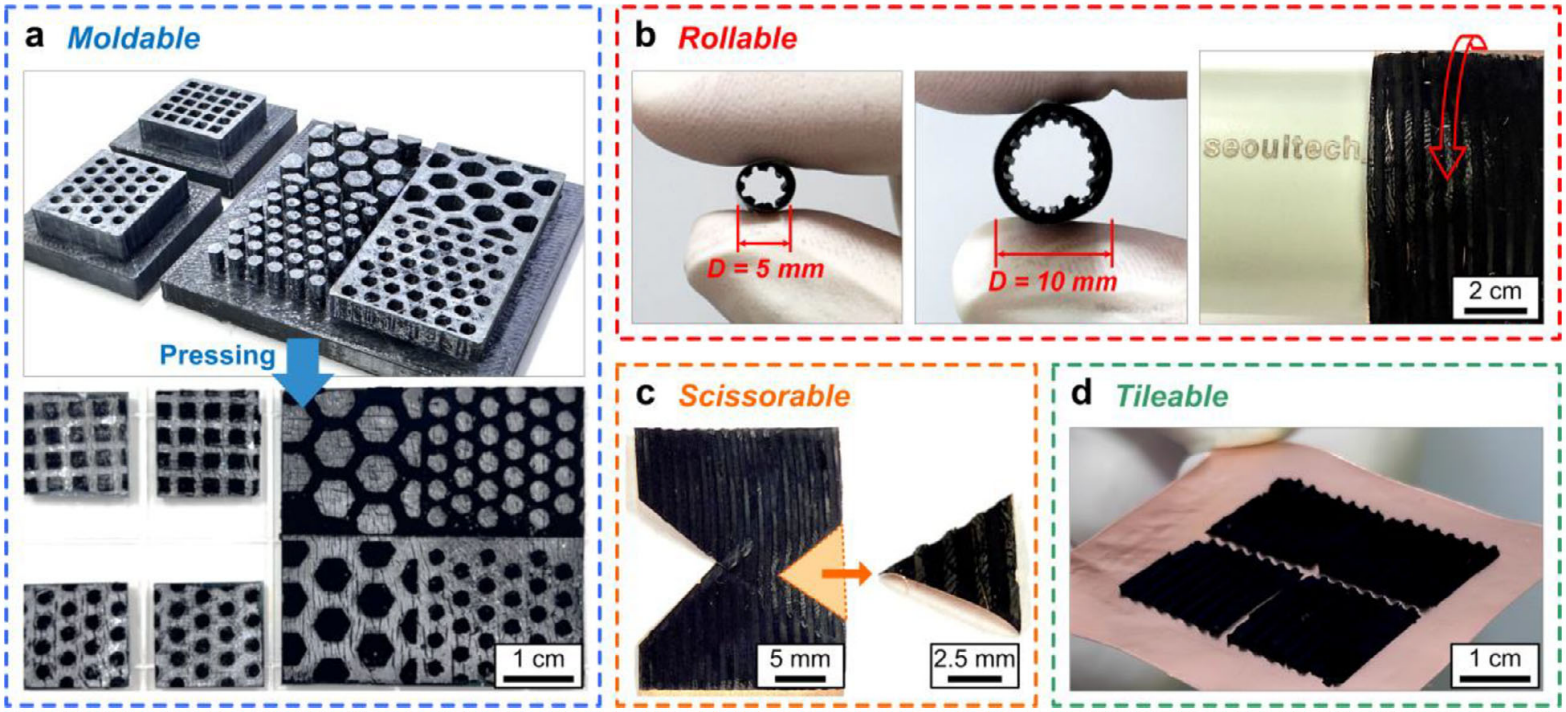
(a) Optical images of various 3D‐printed molds with honeycomb and grid patterns (top) and the resulting patterned VACNT structures (bottom). Optical images exemplyfing the (b) rollable, (c) scissorable, and (d) tileable engineering of the VACNT.

Beyond the mold pressing approach, the VACNT itself possesses several practical handling characteristics that are difficult to achieve with conventional metallic heat sinks. As shown in Figure 5b, the VACNT can be wrapped around cylindrical structures without visible cracking or structural failure. This mechanical compliance originates from the nanoporous VACNT structure [70, 71], which allows the structure to compensate and accommodate large bending deformation. Such mechanical compliance could be advantageous for thermal management of curved or irregularly shaped surfaces where conventional metallic heat sinks are difficult to be installed. The rollable characteristic further presents the potential applicability of the VACNT beyond planar heat sinks, in such forms as a conformal wrapping element for cylindrical heat pipes or a freeform heat management patch attachable to arbitrary surfaces. The VACNT can also be cut into any shapes using common scissors (Figure 5c), allowing rapid and facile geometric customization without complicated post‐processing. Furthermore, individual VACNT heat sinks can be assembled into a tiled configuration to cover larger areas, as exemplified in Figure 5d. This makes the approach well‐suited for passive thermal management in compact electronic devices, ranging from small embedded systems to laptop‐scale platforms where fan‐free, passive cooling solutions are required.

While these results demonstrate the mechanical adaptability of the VACNT into various device‐specifc forms, it should be noted that the compressive force tolerance of the freestanding VACNT, as characterized in Figure S5, is not extremely high. This suggests that, for practical deployment, the structural design and packaging of the host device should be configured such that the VACNT surface is not subjected to direct mechanical contact or repeated physical loading during operation. From this standpoint, the versatility demonstrated above, namely, the ability to roll, cut, and tile the VACNT into diverse geometries (Figure 5b–d) becomes particularly important; it enables the VACNT heat sink to be shaped and positioned to conform to a given device architecture without requiring direct contact‐bearing installation, rather than relying on additional encapsulation or protective treatment to compensate for limited mechanical robustness. In short, the VACNT heat sinks can be adapted to a wide range of device geometries and surface areas in diverse forms and patterns. Some of the actual implementations and performance characterization are currently underway for a successive report.

## 3 Conclusion

In this study, we have developed a VACNT heat sink that directly exploits its multiscale structure for passive multimodal thermal management. The freestanding VACNT, facilely delaminated from its growth substrate by an oxidation process on the catalyst layer, has exhibited competent thermal conductivities of 32–40 W/mK for the growth heights of 500–1500 µm, while retaining mechanical handleability. Upon evaluation on a Raspberry Pi Zero 2 W platform, the 1500 µm VACNT heat sink has decreased the peak steady‐state CPU temperature by 10.7°C relative to the bare CPU operation. The patterned VACNT heat sink has further achieved a 6.7°C lower peak CPU temperature than a commercial aluminum heat sink of the identical pattern geometry, highlighting the unique thermal advantage of the VACNT structure. This advantage is collectively attributed to the superior emissivity (ε ≈ 0.98) of the VACNT surface enabling efficient radiative heat dissipation, and the large effective surface area arising from its nanoporous structure, facilitating convective heat exchange. In addition, the VACNT heat sink has exhibited stable operation over repeated thermal cycles and could be readily patterned into diverse geometries, including fin‐array, honeycomb, and grid structures. Combined with its rollable, scissorable, and tileable characteristics, the VACNT provides exceptional design flexibility for practical and broad thermal management applications. This work presents a promising strategy for passive cooling systems that simultaneously capitalizes thermal conduction, convection, and radiation for next‐generation electronic devices.

## 4 Experimental Section

### 4.1 Growth of VACNT

A SiO_2_ wafer (wet, 3000 Å) was used as the growth substrate. A catalyst layer consisting of Al_2_O_3_ (10 nm) and Fe (2 nm) was sequentially deposited by electron beam evaporation (Sci‐En Tech Co., Ltd., Republic of Korea) under a base pressure of approximately 10^−7^ Torr using a 6 kW sweep. The deposited substrate was cleaved to a proper size (typically 1.5 cm × 1.5 cm) prior to CVD processing. Briefly, after the sample loading in a 2‐inch quartz tube chamber, the catalyst was annealed at 750°C under a gas flow of Ar (300 sccm) and H_2_ (120 sccm) for 7 min. The VACNT growth was subsequently carried out at 750°C under atmospheric pressure with a gas mixture of Ar (300 sccm), H_2_ (120 sccm), and C_2_H_4_ (90 sccm). Growth heights from 100 to 1500 µm were obtained by controlling the duration time of the C_2_H_4_ supply (2 min to 1 h). The detailed process mechanism for the VACNT growth can be found in the literature [38, 39, 40, 41, 42].

### 4.2 Delamination of VACNT

Following the VACNT growth, the oxidation process of the catalyst layer was performed in the same quartz tube furnace at 600°C under a gas flow of Ar (1000 sccm) and air (100 sccm) for 15 to 30 min to help delaminate the VACNT from its growth substrate. Under these conditions, oxygen preferentially reacts with the chemically less stable carbon atoms at the VACNT‐catalyst interface, releasing them as CO_2_, enabling clean mechanical delamination of the VACNT.

### 4.3 Structural Characterization

The growth height of each VACNT sample was measured by using optical microscopy (DVM6, Leica, Germany). The SEM imaging was performed by using a field emission scanning electron microscope (FE‐SEM; SU8010, Hitachi High‐Tech, Japan). The mechanical properties of the VACNT were characterized using a universal testing machine (Instron ElectroPuls E3000LT, Instron, USA).

### 4.4 Thermal Property Characterization

Thermal diffusivity (α) of the VACNT was measured along the longitudinal direction of the CNTs (i.e., from the bottom to the top surface) using laser flash analysis (NETZSCH LFA 457 MicroFlash, NETZSCH‐Gerätebau GmbH, Germany) at room temperature. Specific heat capacity (Cp) was measured by differential scanning calorimetry (NETZSCH STA 449 F3, NETZSCH‐Gerätebau GmbH, Germany) at room temperature, using sapphire as a reference. The density (ρ) of each sample was calculated from its measured dimensions and mass. Thermal conductivity (κ) was derived from the relation κ = α × ρ × C_p_. Measurements were performed for the samples with the growth heights of 500, 1000, and 1500 µm. Raman spectroscopy (Raman microscope, Renishaw plc, UK) was used to evaluate the crystallinity of the VACNT.

### 4.5 Patterning and Cu Deposition

The mold for patterning was fabricated by 3D printing (DP102, Sindoh, Republic of Korea). Cu was deposited onto the VACNT surface by DC magnetron sputtering evaporation (Sci‐En Tech Co., Ltd., Republic of Korea) at 150 W, Ar 100 sccm, 2×10^−2^ torr for deposition times of 5, 10, and 15 min.

### 4.6 Thermal Performance Characterizations

A Raspberry Pi Zero 2 W single‐board computer was used as the heat source platform. A thermal interface material (MT224, MAXTEK, Republic of Korea) with a thermal conductivity of 8 W/mK and a thickness of 0.3 mm was attached between the CPU and the VACNT heat sink. VACNT heat sinks of various heights (100, 500, 1000, and 1500 µm) were transferred on the TIM layer under a compressive force of 5 N. The CPU temperature was monitored using the onboard temperature sensor. Thermal imaging was performed using a thermal image camera (E6XT, FLIR Systems, USA). The camera emissivity setting used for infrared thermal images was 0.90 for the TIM surface, 0.90 for the black‐anodized aluminum heat sink, and 0.98 for all VACNT surfaces. Since measuring the aluminum surface at its literature emissivity (ε ≈ 0.05) resulted in unstable readings due to strong reflection, the camera emissivity was set to 0.1 for the aluminum heat sinks to obtain a stable temperature reading.

Peak steady‐state CPU temperature was recorded at each cycle to assess performance consistency. All measurements were conducted at room temperature (∼25°C). Full‐load conditions were achieved by utilizing all four CPU cores simultaneously via a stress test. The cyclic thermal performance was evaluated over 50 heating and cooling cycles under full‐load CPU operation, with each cycle consisting of 15 min of full‐load heating followed by 15 min of cooling.

### 4.7 Demonstration of Practical Applications

For the rollable demonstration, the VACNT was transferred onto a commercial adhesive tape (Scotch Magic Tape 810D, 18 mm × 20 m, 3 m, USA) and compressed using a mold to form a fin array structure. The resulting structure was then manually rolled around cylindrical structures with diameters of 5 and 10 mm to demonstrate its mechanical flexibility and structural integrity under various bending deformations. For the scissorable demonstration, the VACNT was transferred onto a Cu tape (Copper tape #1181, USA) and subsequently cut using scissors. For the tileable demonstration, four individual VACNTs were transferred onto a Cu tape of the same type and arranged in a tiled configuration to form a larger continuous heat sink array, demonstrating the scalability of the VACNT heat sink.

## Author Contributions

**Jongwon Park**: conceptualization, methodology, software, data curation, validation, writing – original draft, visualization, formal analysis. **Kwangjun Kim**: conceptualization, writing – original draft, visualization, methodology. **Jimin Lee**: software, data curation, methodology. **Jong G. Ok**: investigation, data curation, writing – review and editing, project administration, resources, supervision, funding acquisition.

## Funding

This research was supported by grants from the National Research Foundation of Korea (NRF‐2022M3C1A3081178, RS‐2024‐00413607, and RS‐2024‐00449882 (Ministry of Science and ICT), and NRF‐2022R1I1A2073224 (Ministry of Education)) and the Commercialization Promotion Agency for R&D Outcomes (RS‐2026‐25537291) funded by the Korean Government.

## Conflicts of Interest

The authors declare no conflicts of interest.

## Supporting information




**Supporting File**: advs78540‐sup‐0001‐SuppMat.docx.

## Data Availability

The data that support the findings of this study are available from the corresponding author upon reasonable request.
