# Supplementary material for: Multiscale Freestanding Vertically Aligned CNT Heat Sink Enabling Multimodal Heat Dissipation by Conduction, Convection, and Radiation
Source: Adv Sci (Weinh). 2026 Nov 3:e78540. Online ahead of print. doi: 10.1002/advs.78540 (PMC13731875; doi:10.1002/advs.78540)
Supplement: Supplementary file 1 [file ADVS-9999-e78540-s001.docx]

Supporting Information

Multiscale freestanding vertically aligned CNT heat sink enabling multimodal heat dissipation by conduction, convection, and radiation

Jongwon Park^1,†^, Kwangjun Kim^1,†^, Jimin Lee^2^, and Jong G. Ok^1,3,^*

^1^ Department of Automotive Engineering, Seoul National University of Science and Technology, 232, Gongneung-ro, Nowon-gu, Seoul 01811, Republic of Korea

^2^ Department of Mechanical and Automotive Engineering, Seoul National University of Science and Technology, 232, Gongneung-ro, Nowon-gu, Seoul 01811, Republic of Korea

^3^ School of Mechanical Systems Engineering, Seoul National University of Science and Technology, 232, Gongneung-ro, Nowon-gu, Seoul 01811, Republic of Korea

^†^Jongwon Park and Kwangjun Kim equally contributed to this work.

*Corresponding author: Dr. Jong G. Ok, jgok@seoultech.ac.kr, Tel. +82-2-970-9012

**Figure S1.** (a) Scanning electron microscopy (SEM) images of the annealed Fe catalyst nanoparticles on the Al_2_O_3_/SiO_2_ substrate prior to VACNT growth and (b) its binarized image used for quantitative particle analysis. The average nanoparticle diameter and areal density were determined to be 17.3 nm and 7.44 × 10^10^ /cm^2^, respectively.Optical top-view images of the substrates (1.5 × 1.5 cm^2^) (c) before and (d) after the VACNT growth. (e) Optical side-view images of the grown VACNTs with the growth height of 1 mm. The mass of a representative 1.5 × 1.5 cm^2^ VACNT sample with the growth height of 1 mm was measured to be 87.0 mg, from which the density was determined to be 386.5 mg/cm^3^.

**Figure S2.** Differential scanning calorimetry (DSC) curves of the VACNT sample and the sapphire reference standard (STD 25 mm DSC), and the resulting specific heat capacity (C_p_) of VACNT as a function of temperature. The C_p_ value at 25 °C was determined to be 1.254 J/gK which was used for the thermal conductivity calculation in this study.

**Figure S3.** (a-c) Schematics of the VACNT heat sink fabrication procedure, showing the sequential steps: (a) TIM attachment, (b) VACNT transfer, and (c) mold pressing for fin array patterning.

**Figure S4.** Infrared thermal images of the top and lateral sidewall surfaces under full-load steady state. (a) Top view of the TIM only condition. (b-g) Top views of the VACNT heat sinks with the growth heights of (b) 100 µm, (c) 500 µm, (e) 1000 µm, and (g) 1500 µm. Side views of the VACNT heat sinks with the growth heights of (d) 500 µm, (f) 1000 µm, and (h) 1500 µm. The sidewall temperature of the 100 µm VACNT was not resolved due to its small height. These values were used to calculate Q and R_TIM-air_ in Table 1 and Table 2.

**Figure S5.** Compressive stress-strain curve of the 1000 µm VACNT, measured using a universal testing machine. The applied force required to achieve the target fin-to-base height ratio of 2:1 was determined from this curve and used for the mold-pressing patterning process.

**Figure S6.** Infrared thermal images of the top and lateral sidewall surfaces of the aluminum and patterned VACNT heat sinks under full-load steady state. Top views of (a) the Al heat sink (1.5 mm), (c) the Al heat sink (6 mm), and (e) the patterned VACNT heat sink. Side views of (b) the Al heat sink (1.5 mm), (d) the Al heat sink (6 mm), and (f) the patterned VACNT heat sink. The top-surface temperatures shown in (a, c, e) were used to calculate Q and R_TIM-air_ in Table 3. The sidewall temperatures shown in (b, d, f) are provided for reference only.

**Figure S7.** Thermal performance of the black-anodized Al heat sink (6 mm) compared with the VACNT heat sink. (a) Optical and infrared (inset) images of the Raspberry Pi with a black-anodized aluminum heat sink (6 mm) under full load conditions. (b) CPU temperature profiles and (c) summary of CPU temperatures under idle and full load conditions.

**Figure S8.** Top-view optical images of the 1500 µm VACNT (a) before and (b) after Cu sputter deposition. Cross-sectional SEM image of the (c) 5 min, (d) 15 min Cu-deposited VACNT, where it could be observed that the nanoporous structure becomes increasingly filled with Cu deposition time. (e) CPU temperature profiles and (f) summary of CPU temperatures under idle and full load conditions.

**Figure S9.** SEM images of the 500 µm VACNTs with (a) low, (b) normal, and (c) high nanoporosity, fabricated by varying the catalyst annealing duration prior to growth; the normal-porosity VACNT is identical to that used throughout this work (Figure 1h). (d) CPU temperature profiles during idle and full-load operation and (e) summary of CPU temperatures under idle, full-load average, and steady-state conditions, for the three VACNT conditions.
